# A phase-retrieval toolbox for X-ray holography and tomography

**DOI:** 10.1107/S1600577520002398

**Published:** 2020-04-14

**Authors:** Leon M. Lohse, Anna-Lena Robisch, Mareike Töpperwien, Simon Maretzke, Martin Krenkel, Johannes Hagemann, Tim Salditt

**Affiliations:** a Institut für Röntgenphysik, Universität Göttingen, Germany; b Deutsches Elektronen-Synchrotron, Hamburg, Germany

**Keywords:** X-ray imaging, propagation-based phase contrast, holography, phase retrieval, tomography

## Abstract

Propagation-based phase-contrast X-ray imaging is by now a well established imaging technique, which – as a full-field technique – is particularly useful for tomography applications. Since it can be implemented with synchrotron radiation and at laboratory micro-focus sources, it covers a wide range of applications. A limiting factor in its development has been the phase-retrieval step, which was often performed using methods with a limited regime of applicability, typically based on linearization. In this work, a much larger set of algorithms, which covers a wide range of cases (experimental parameters, objects and constraints), is compiled into a single toolbox – the *HoloTomoToolbox* – which is made publicly available. Importantly, the unified structure of the implemented phase-retrieval functions facilitates their use and performance test on different experimental data.

## Abstract 2

This article describes scientific software made publicly available for phase retrieval in X-ray propagation imaging. The package is called *HoloTomoToolbox* and comprises a set of algorithms for phase retrieval in the direct-contrast regime, as well as in the holographic regime, which is used for high-resolution cone-beam synchrotron recordings. Further to this, a set of routines is included to facilitate the conversion of the 2D phase projections in a 3D tomogram.

## 1. Introduction   

**Figure 1:**
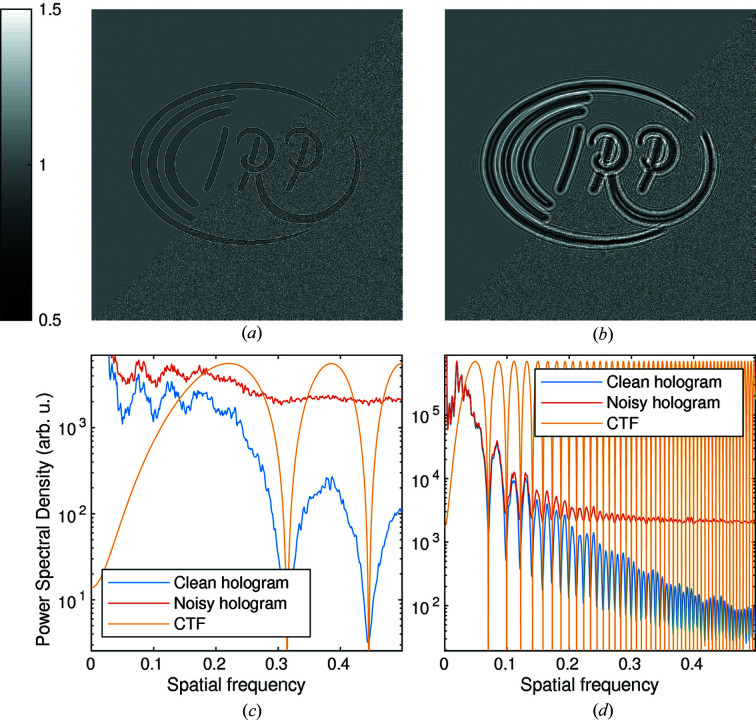
Image formation and CTF. A common wavefield (phase shift 0.5 rad, β/δ = 0.05) is propagated with 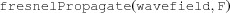 to different Fresnel numbers *F*. A shot noise of 50 photons pixel^−1^ is introduced (lower-right half) to the holograms with 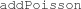 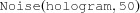. (*a*) Hologram in the direct-contrast regime (*F* = 0.1). (*b*) Hologram in the holographic regime (*F* = 0.005). (*c*, *d*) Radially averaged power spectral density, obtained by 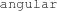 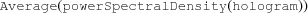, of the noisy and clean holograms in both regimes. The CTF (weak object approximation) is also drawn. Similar graphs can be quickly produced with 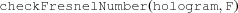 to verify the Fresnel numbers of experimental data. The wavefield was obtained by exponentiating the phantom 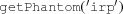.

For imaging complex samples at high resolution, hard X-rays offer a unique potential because of their small wavelength and high penetration depth. In classical radiography, contrast is based on absorption of the beam when passing through an object. However, in order to resolve the inner structure of weakly absorbing materials and biological specimens at sufficient contrast, in addition to absorption, the phase information, *i.e.* the phase difference in the X-ray wave induced by different phase velocities in a partially coherent wavefront, has to be exploited. Propagation-based phase-contrast X-ray imaging (PBI) has proven to be a suitable method to make this phase difference visible via the conversion of phase information into measurable intensity patterns by ways of self-interference of the beam between object and detector, *i.e.* by free-space propagation (Wilkins *et al.*, 1996 ▸; Cloetens *et al.*, 1999 ▸; Paganin, 2006 ▸; Nugent, 2011 ▸). PBI has also been compared with other phase-contrast methods such as analyser or grating-based techniques and is particularly well suited for high-resolution imaging (Zanette *et al.*, 2013 ▸; Lang *et al.*, 2014 ▸). Image formation in PBI is well understood. The measurable intensity pattern depends on the length scales of propagation distance *z*, photon wavelength λ and sample structure *A*. The dependence on these parameters can be summarized in terms of the Fresnel number *F*_(*A*)_ = *A* ^2^/(*z*λ) (associated with the object scale *A*). Depending on the Fresnel numbers 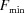 and 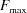 associated with the smallest and largest relevant image structures (*e.g.* the size of one pixel and of the full field-of-view, respectively), different imaging regimes can be distinguished: the direct-contrast regime (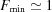), in which phase-contrast effects are visible as edge enhancements but contrast transfer for low spatial frequencies is weak [see Fig. 1(*a*) ▸]; the holographic regime (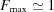, 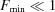), in which multiple interference fringes are visible and coarser image structures exhibit high contrast transfer [see Fig. 1(*b*) ▸]; and the far-field regime (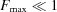), where the intensity pattern is essentially given by the squared modulus of the Fourier transform of the wavefield behind the sample (Fraunhofer far-field), without any interference with the primary beam. An important advantage of PBI is the high number of resolution elements which can be imaged in parallel with a single acquisition without any scanning, making it extremely suitable for tomography. Hence in this work, PBI is always associated with primarily 3D applications.

PBI is predominantly carried out with synchrotron radiation (SR) because of the relatively high degree of spatial coherence offered at these sources. High resolution at the nano­scale is achieved by focusing SR to a secondary source and then exploiting the cone-beam geometry (Mokso *et al.*, 2007 ▸; Bartels *et al.*, 2015 ▸) and the Fresnel scaling theorem (Paganin, 2006 ▸). A wide range of dedicated beamlines are now available for parallel-beam PBI (see, for example, Weitkamp *et al.*, 2010 ▸; Longo *et al.*, 2019 ▸; Wang *et al.*, 2001 ▸; Schell *et al.*, 2013 ▸) as well as – at a somewhat smaller scale and number – for cone-beam PBI based on nano-focused undulator radiation (da Silva *et al.*, 2017 ▸; Wilde *et al.*, 2016 ▸; Lovric *et al.*, 2016 ▸; Salditt *et al.*, 2015 ▸). In addition, a new generation of micro-focus X-ray sources, with increased flux and improved spatial coherence (Hemberg *et al.*, 2003 ▸), has enabled the implementation of PBI and computed tomography at the micrometre scale (microCT) in the laboratory (Bartels *et al.*, 2013 ▸; Vågberg *et al.*, 2015 ▸; Larsson *et al.*, 2016 ▸).

As for other coherent X-ray imaging techniques, a central challenge for PBI is the reconstruction of the projected phase images from the measured interference patterns, *i.e.* the phase-retrieval step. There exist efficient algorithms to perform this task in practice, either by a direct single-step inversion based on various approximations or by iterative techniques based either on minimizing an error metric (Maretzke *et al.*, 2016 ▸; Davidoiu *et al.*, 2011 ▸) or in the framework of projection algorithms (Luke *et al.*, 2002 ▸; Marchesini, 2007 ▸). While powerful software packages have been published for far-field coherent imaging, notably for coherent diffractive imaging (Maia, 2008 ▸) and ptychography (Marchesini *et al.*, 2016 ▸; Enders & Thibault, 2016 ▸), publicly available phase-retrieval and reconstruction software for PBI is still sparse (Weitkamp *et al.*, 2011 ▸), limited to the direct-contrast regime of PBI. Here, the commonly used technique is the single material object algorithm (SMO algorithm) (Paganin *et al.*, 2002 ▸). Based on this, different research teams at PBI beamlines have developed elaborate codes which have also resulted in actively maintained open-source frameworks (Weitkamp *et al.*, 2011 ▸; da Silva, 2019 ▸; Gürsoy *et al.*, 2014 ▸). Among them, *TomoPy* (Gürsoy *et al.*, 2014 ▸) is an outstanding example of a framework for tomography that also includes phase retrieval for the direct-contrast regime. However, it is neither suitable for the holographic imaging regime with SR nor for the low coherence setting of laboratory microCT, which requires a different set of reconstruction algorithms. Further progress and development of imaging techniques could benefit tremendously from commonly accessible code which is more general with respect to the phase-retrieval approach applicable for different imaging regimes, requirements of probe coherence and object constraints. In addition to processing of experimental data, a framework should also allow simulating experiments beforehand and finding appropriate phase-retrieval settings. In terms of algorithms, many schemes established for the far-field can be adapted to PBI by exchange of the propagators. Yet, since the mathematical structure of the phase-retrieval problem changes its nature when applied to the near-field setting (see, for example, Maretzke, 2015 ▸), it has to be kept in mind that the same algorithm can behave quite differently in different imaging regimes.

In this work, we present a software toolbox containing a collection of MATLAB functions developed by and implemented in our group over several years, which allows for phase retrieval in both the direct-contrast and holographic regimes. It originated from the need to facilitate analysis for data obtained at the holotomography endstation GINIX, which is installed at the P10 beamline at PETRA III located at DESY (Salditt *et al.*, 2015 ▸), as well as at the emerging laboratory-based setups which are now available for PBI in the direct-contrast regime.

**Figure 2:**
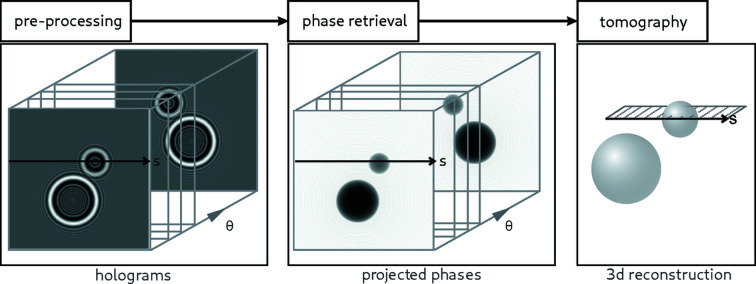
General workflow of image pre-processing, followed by phase retrieval and finally tomographic reconstruction. Note that some 3D phase-retrieval methods either combine steps 2 and 3 or interchange them, exploiting the linearity of the contrast formation (weak object approximation).

The rising number of internal and external users at these setups has raised the demand for a self-contained toolbox covering the entire data-analysis process illustrated in Fig. 2 ▸, including a collection of phase-retrieval algorithms. For that purpose, the toolbox has recently undergone a thorough revision. The function names and interfaces were harmonized and the code was refactored and partially rewritten from scratch, exploiting more recent features of MATLAB and eliminating redundancy. In addition, detailed documentation was added. The toolbox, called *HoloTomoToolbox*, is now presented in this work as an overview and at the same time made publicly available under the GPLv3 license. *HoloTomoToolbox* is available under https://gitlab.gwdg.de/irp/holotomotoolbox including documentation and worked-through examples. The name accounts for the fact that the applications include in particular the holographic regime, which is not yet well covered, and that we assume that the vast majority of applications will be 3D.

Note that for the 3D tomographic reconstruction, *HoloTomoToolbox* interfaces the efficient and versatile *ASTRA* toolbox (van Aarle *et al.*, 2015 ▸, 2016 ▸) that, in particular, provides a fast implementation of the Feldkamp–Davis–Kress (FDK) algorithm (Feldkamp *et al.*, 1984 ▸) for cone-beam tomography. Here we do not want to give a comparison of algorithms for specific experimental conditions and parameters, since this has partly been the topic of previous work (see, for example, Burvall *et al.*, 2011 ▸; Krenkel *et al.*, 2017 ▸). Rather, we want to provide a user-friendly collection of different phase-retrieval approaches suitable for real-world applications. This also accounts for the fact that there is no optimal method covering all cases but that instead each configuration requires a careful consideration of the different alternatives.

## 2. Propagation-based phase-contrast X-ray imaging   

To illustrate the various regimes and assumptions made in phase-retrieval algorithms, we will briefly cover the underlying forward models [see also Maretzke (2019 ▸) and references therein]. Image formation of fully coherent PBI is modeled by formulating the measured intensity pattern *I* as a function of projected phase (assuming that the projection approximation holds) 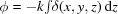 and absorption 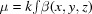 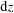 where *n* = 1 − δ + *i*β is the refractive index, *z* is the optical axis and *k* is the wave number as 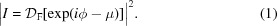
Here, 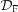 denotes Fresnel propagation of the wavefield in the object plane 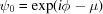 to the detector, governed by the Fresnel number *F*. Phase retrieval consists of reconstructing the phase ϕ (and absorption μ) from data of the form (1). To facilitate this task, additional simplifying assumptions can be made, as described in the following.

### 2.1. Weak object approximation   

One of the most widely used approximations in near-field phase retrieval is the assumption that the sample interacts sufficiently weakly with the X-rays such that the relation (1) can be linearized with respect to the phase and absorption images ϕ and μ. Neglecting higher-order contributions in the components of the refractive index and rearranging, this leads to the well known contrast-transfer function (CTF) model (Guigay, 1977 ▸), 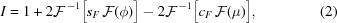
where *s*_*F*_ and *c*_*F*_ denote the phase and absorption CTFs, and 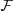 denotes the Fourier transform. The linearization in (2) has a surprisingly large regime of validity (Turner *et al.*, 2004 ▸) but can also fail completely in the presence of large phase gradients (Hagemann *et al.*, 2018 ▸).

### 2.2. Pure phase and single-material approximation   

Typically, the absorption contrast μ is much weaker than the phase ϕ. Depending on the optical constants and size of the object, it can even be justified to neglect absorption altogether, *i.e.* to assume pure phase contrast μ = 0, and hence use this constraint for phase retrieval. If the absorption μ cannot be neglected, but is expected to scale with the local electron density, a popular constraint is to set it proportional to the phase, μ/ϕ = β/δ = *c*_β/δ_ = constant. This single-material approximation strictly holds only for objects where the stochiometry of elements (not their density) is homogeneous throughout the object. The obvious algorithmic benefit of either pure phase contrast or single-material assumptions is that only one image (ϕ) instead of two (ϕ and μ) have to be recovered from the measured data.

### 2.3. Linearized transport of intensity – the direct-contrast regime   

In the direct-contrast regime, the governing Fresnel numbers are large enough that the model in equation (1) can be well approximated by linearization with respect to 1/*F*. This is equivalent to a frequently used model based on the transport-of-intensity (TIE) equation (see, for example, Teague, 1983 ▸), 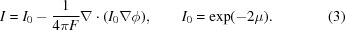



## 3. Overview of the toolbox   

The *HoloTomoToolbox* extends the built-in functionalities of MATLAB to enable the entire data-analysis workflow of a typical holotomography measurement. First, reading and pre-processing the raw data to obtain clean and aligned holograms; second, the phase-retrieval step from holograms to phase maps; third, the tomographic reconstruction from 2D projections to 3D volumes; and, finally, post-processing of the 3D volume (see Fig. 2 ▸).

Besides the main functionalities, the toolbox also contains a large number of convenience functions, *e.g.* for simulation of PBI data and general image processing. In addition, the toolbox provides exemplary experimental holograms, acquired at laboratory and synchrotron setups by our group, including all the 2D PBI examples presented in Section 4. *HoloTomoToolbox* is built on top of a number of low-level functions to work around various shortcomings of MATLAB and allow for more efficient and clearer code. Prominent examples are the functions 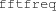 and 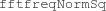, inspired by *NumPy*, which create a (properly shifted) grid in Fourier frequencies corresponding to the sampling points of an *n*-dimensional fast Fourier transform and its (Euclidean) norm square, respectively. They simplify the definition of Fourier-space filters tremendously and are used in virtually all phase-retrieval functions.

The main aims of the revision were to make the code clearer and easier to read, but also making it faster, avoiding redundancy and using MATLAB’s built-in algorithms whenever possible.

In the same spirit, the function interfaces were designed to be as easy to use as possible, while at the same time exposing sufficient control knobs. On the one hand, this requires as few (mandatory) parameters as possible while including optional parameters that have meaningful default values, and on the other hand this requires meaningful and consistent parameter names. Because of MATLAB’s syntax for function definitions, it is impossible to have multiple optional parameters and provide default values in a simple user-friendly way. For this reason, many functions in *HoloTomoToolbox* optionally accept a structure array 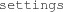 as their last argument to pass extra parameters. All omitted parameters are filled with their default values. The default values can be obtained by calling the function with no arguments and can also be found in the documentation.

All phase-retrieval functions in the toolbox have a unified interface so that different algorithms for a specific task can be easily tested and compared. Every function comes with detailed documentation and simple examples are included to help non-experts in getting started. An important concept of the toolbox is to use pixel units and the unit-less Fresnel number *F* for all tasks. We note that we can only give a brief overview with references here, while detailed documentation with examples for most functions is available online (https://gitlab.gwdg.de/irp/holotomotoolbox).

### 3.1. Pre-processing: data input and alignment   

Various functions are provided for reading the acquired detector data, complementing MATLAB’s built-in 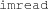.

Apart from the obvious corrections by subtraction of dark images or division by the flat field (intensity profile of the illumination) or filtering of low-frequency artifacts, which can trivially be accomplished using MATLAB’s built-in functions, other pre-processing steps are more involved, *e.g.* the removal of hot pixels/outliers or stripes. The toolbox also provides a collection of other recurring image-processing tasks, such as cropping, padding and windowing of 2D or 3D data.

A major part of pre-processing is image alignment. For this reason, the toolbox provides general methods for image registration of shifts and rotations based on Guizar-Sicairos *et al.* (2008 ▸), and methods to shift, rotate and magnify images. In addition, it provides functions for more specific tasks, such as the automatic alignment of holograms acquired at different defocus/propagation distances or the automatic alignment of the tomographic axis.

### 3.2. Phase retrieval   

A unique feature of the toolbox is the variety of implemented methods for near-field phase retrieval. All methods require (dark- and flat-field corrected) holograms and, depending on the regime, the corresponding Fresnel numbers as input. Free-space propagation is computed assuming a fully coherent plane-wave illumination. In practice, however, the methods still perform well for partially coherent data (Hagemann & Salditt, 2018 ▸) and turn out to be much more robust than far-field methods.

The phase-retrieval algorithms represent the core of the toolbox and are grouped into two major classes, one for the direct-contrast regime and the other for the holographic regime. Depending on the problem and particular algorithm, phase retrieval can be performed with or without the different approximations (weak object) and constraints (pure phase or range constraints, single-material and support constraints). The methods for the holographic regime encompass both single-step reconstruction algorithms, which are computationally faster, and iterative ones, which require less assumptions (in particular, no weak object) and provide more flexibility in terms of imposing *a priori* constraints. By performing computations on a graphics processing unit (GPU), reconstruction with iterative algorithms also becomes feasible for tomography applications.

Table 1 ▸ lists the three algorithms implemented for the direct-contrast regime [SMO, modified Bronnikov algorithm (MBA), Bronnikov aided correction (BAC)], with corresponding original references and function names. A comparison for laboratory microCT applications of the three approaches has been presented in the work of Töpperwien *et al.* (2016 ▸), which also gives a guideline on how to choose the respective regularization parameters. Further help and example scripts are provided in the documentation.

Table 2 ▸ lists the implemented algorithms for phase retrieval in the holographic regime. The CTF implementation follows very closely the seminal work by Cloetens and co-workers (Cloetens *et al.*, 1999 ▸; Turner *et al.*, 2004 ▸; Zabler *et al.*, 2005 ▸). It has been the working horse for holographic phase retrieval for nearly two decades but the trend is turning to iterative methods nowadays to overcome the limitations imposed by the weak object approximation. In our toolbox, the implementation of CTF-based phase retrieval is improved by the possibility to include additional constraints as a support or by the positivity of the reconstructed phase. The extension of the TIE approach to the holographic regime (HoloTIE algorithm) is also based on multiple-distance acquisitions as CTFs, but here very closely spaced to approximate the intensity derivative with propagation distance *z*. It has been introduced in the work of Krenkel *et al.* (2013 ▸, 2017 ▸) and offers a suitable alternative for cases where no object constraint is available, *i.e.* for objects which are extended, not pure phase and not single material. The nonlinear Tikhonov algorithm is based on a fully nonlinear phase-contrast model inverted with Tikhonov regularization (unpublished work by Maretzke) and will be discussed in a future article.

Next, the table lists the iterative projection algorithms [alternating projections (AP), relaxed averaged alternating reflections (RAAR) and modified hybrid input output (mHIO)] again with the corresponding function names and references, referring to their first use for PBI and representing the starting points for the toolbox implementation. These algorithms are based on alternating application of projection-operators in the object and data space corresponding to constraints in the respective spaces and offer a similarly large regime of applicability and flexibility in terms of constraints. Similar to the implementation in the work of Luke (2012 ▸), the toolbox provides the projector classes as well as abstract algorithms that can be freely combined to try out new algorithm-constraint combinations. In addition, the most useful and robust combinations (*e.g.* the reconstruction of a pure-phase object and support constraints) are provided ready to use with meaningful default parameter values.

Finally, two iterative algorithms for simultaneous phase retrieval and tomographic reconstruction (3D phase retrieval) are provided: iterative reprojection phase retrieval (IRP) and the more recent 3D CTF method. These fully 3D approaches enable improved reconstruction quality as demonstrated in the work of Ruhlandt & Salditt (2016 ▸), yet at the cost of increased computation time.

### 3.3. Tomographic reconstruction   

The toolbox contains easy-to-use wrappers for GPU-based tomographic reconstruction methods implemented in the *ASTRA* toolbox (van Aarle *et al.*, 2015 ▸, 2016 ▸), including filtered back projection, the simulatenous iterative reconstruction technique for parallel-beam tomography and the FDK algorithm for cone-beam data. Additionally, wrappers for tomographic (unfiltered) forward- and back-projections are provided, which can be used for data simulation or as building blocks for iterative algorithms.

### 3.4. Post-processing (ring removal)   

Because of the varying sensitivities of detector pixels or dust particles on the sensors, tomographic reconstructions often suffer from so-called ring artifacts. The toolbox offers functions to remove concentric ring structures from the recovered images using established algorithms (Ketcham, 2006 ▸; Münch *et al.*, 2009 ▸).

## 4. Examples   

**Figure 3:**
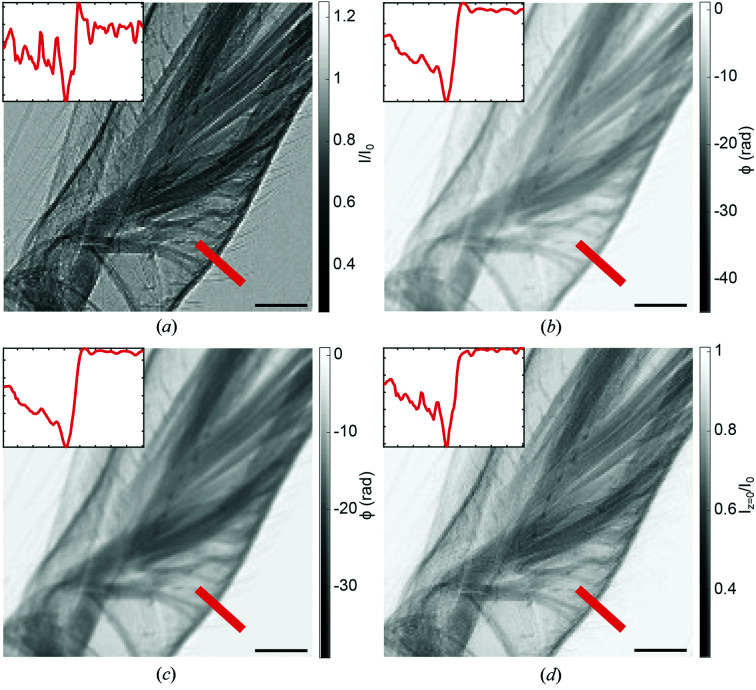
Phase retrieval in the direct-contrast regime. Example data showing a spider leg (zoom), recorded at a laboratory microCT setup with a rotating anode jet source. (*a*) A raw projection without the application of a phase-retrieval algorithm. The inset shows a line profile along the red line. The typical edge enhancement can be seen visually in the projection as well as in the profile. (*b*) A reconstructed phase using the SMO method with a β/δ ratio of 0.015. The edge enhancement is compensated, as can be seen in the projection image and the line profile, but the reconstructed phase seems blurred in comparison with the raw data. (*c*) A reconstructed phase using the MBA algorithm with a regularization parameter of 0.04. As in the SMO case, the edge enhancement can be reduced but also here the reconstruction seems blurred compared with the raw data. (*d*) A reconstructed intensity distribution in the object plane using the BAC algorithm. The edge enhancement is again removed by applying the reconstruction algorithm but, in contrast to the SMO and MBA methods, the reconstructed image is almost as sharp as the raw data. Data are supplied in the generators folder and can be loaded in via 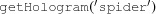. The scale bar represents 100 µm Data taken from the work of Töpperwien *et al.* (2016 ▸).

This section lists reconstructions obtained with *HoloTomoToolbox* for typical use cases. As a first example, laboratory microCT applications are presented where phase effects are observed in the direct-contrast regime. A particularly well suited approach for PBI at such sources is to use the inverse geometry at low magnification, *i.e.* a source-to-sample distance much larger than the sample-to-detector distance and a high-resolution detector. Here, the system resolution is mainly determined by the point-spread function of the detector, and partial spatial coherence is sufficient to observe phase effects. As shown in the work of Töpperwien *et al.* (2016 ▸), a suitable phase-reconstruction strategy follows BAC (De Witte *et al.*, 2009 ▸), since this approach is robust with respect to the non-ideal beam conditions at compact X-ray sources, namely low spatial coherence and large bandwidth. A comparison of different phase-retrieval approaches for the direct-contrast regime is given in Fig. 3 ▸. The example shows a detailed view of an iodine stained cobweb spider leg, with the normalized projection, and the corresponding reconstructions by the MBA (Groso *et al.*, 2006 ▸), the SMO (Paganin *et al.*, 2002 ▸) and the BAC approach (De Witte *et al.*, 2009 ▸). Only BAC provides a reconstruction with sharpness comparable with the raw data. This affects in particular the resolution of the 3D volume data obtained from BAC-reconstructed projections, as demonstrated in the work of Töpperwien *et al.* (2016 ▸). Note that since BAC is in essence a physically motivated smoothing operator, it enhances the signal-to-noise ratio compared with projections recorded in the direct-contrast regime while at the same time exploiting the pronounced contrast of the edge-enhancement regime.

**Figure 4:**
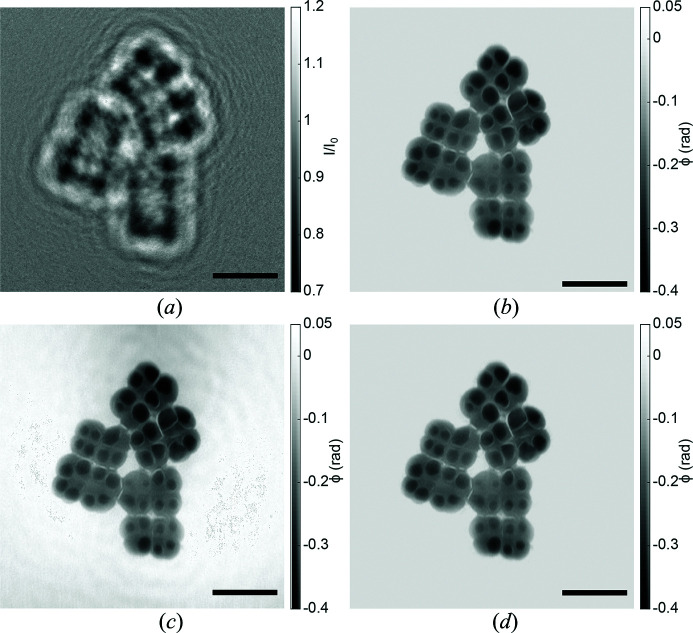
Phase retrieval in the holographic regime. (*a*) Hologram. (*b*) RAAR with support- and maximum-phase constraint. (*c*) CTF without constraints; parameters: lim1 = 5 × 10^−4^, lim2 = 1 × 10^−2^, β/δ = 0. (*d*) CTF with support- and maximum-phase constraint. Data are supplied in the generators folder and can be loaded via 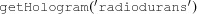. The scale bar represents 5 µm. Data taken from the work of Bartels *et al.* (2015 ▸).

**Figure 5:**
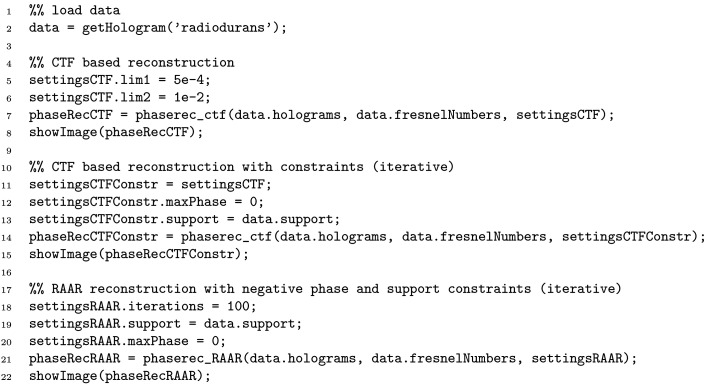
Example code that loads supplied example holograms and reconstructs the phase shift with different phase-retrieval algorithms. The phase-retrieval functions share a common interface and only require a minimal number of parameters. For more examples refer to the online documentation.

Fig. 4 ▸ illustrates phase retrieval in the holographic regime for the case of high-resolution (cone-beam) SR data, showing (*a*) the flat-field-corrected hologram of freeze-dried *Deinococcus radiodurans* cells [single-distance recording with 13.8 keV at the GINIX setup with a sub-20 nm secondary source (Bartels *et al.*, 2015 ▸)], and (*b*)–(*d*) different phase reconstructions with­out (left side) and with (right side) support constraint. The source code required to produce the shown reconstructions is listed in Fig. 5 ▸.

## 5. Conclusions   

The phase problem in the near-field holographic setting has been the subject of intensive research for more than two decades and significant progress has been achieved. However, the range of experimental parameters, the object’s optical properties and the variety in prior knowledge require a set of phase-retrieval algorithms instead of a single tool. To this end, we have presented *HoloTomoToolbox*, which we hope may prove useful for other groups and provides a reference for further work. To our knowledge, this is the first published and free collection of near-field phase-retrieval methods for different imaging regimes. It facilitates phase-retrieval applications at current instruments, both with SR and microCT. We also suggest to use *HoloTomoToolbox* for educational purposes to demonstrate/experiment with near-field phase-retrieval methods in classroom or workshop tutorials. Finally, it may also inspire further development, in particular when it comes to more efficient codes and more rigorous GPU implementations.

## Figures and Tables

**Table 1:** List of the implemented phase-retrieval algorithms for the direct-contrast regime together with the original references

Name	Function name	References
Paganin / Single Material Object (SMO)	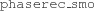	Paganin *et al.* (2002 ▸)
Modified Bronnikov (MBA)	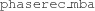	Groso *et al.* (2006 ▸)
Bronnikov Aided Correction (BAC)	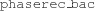	De Witte *et al.* (2009 ▸)

**Table 2:** List of the implemented phase-retrieval algorithms for the holographic regime The given references correspond to the first use in the near-field regime (to the best of our knowledge). For original work on possible previous applications of the algorithms, see the references therein.

Name	Function name	References
Contrast-Transfer-Function (CTF)	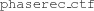	Cloetens *et al.* (1999 ▸)
Nonlinear Tikhonov	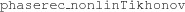	Maretzke (unpublished)
Holo-TIE (HoloTIE)	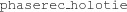	Krenkel *et al.* (2013 ▸)
Alternating Projections (AP)	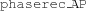	Hagemann *et al.* (2018 ▸)
Relaxed Averaged Alternating Reflections (RAAR)	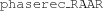	Luke (2005 ▸)
modified Hybrid Input Output (mHIO)	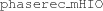	Giewekemeyer *et al.* (2011 ▸)
		
3D phase retrieval (tomographic)		
Iterative Reprojection Phase Retrieval (IRP)	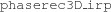	Ruhlandt *et al.* (2014 ▸)
3D-CTF	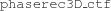	Maretzke (unpublished)
